# Omarigliptin Mitigates 6-Hydroxydopamine- or Rotenone-Induced Oxidative Toxicity in PC12 Cells by Antioxidant, Anti-Inflammatory, and Anti-Apoptotic Actions

**DOI:** 10.3390/antiox11101940

**Published:** 2022-09-28

**Authors:** Noha A. Gouda, Jungsook Cho

**Affiliations:** College of Pharmacy and Integrated Research Institute for Drug Development, Dongguk University-Seoul, 32 Dongguk-ro, Ilsandong-gu, Goyang 10326, Gyeonggi, Korea

**Keywords:** omarigliptin, oxidative stress, nuclear factor erythroid 2-related factor 2, heme oxygenase-1, nuclear factor-kappaB, apoptosis, Parkinson’s disease, drug repurposing, PC12 cells

## Abstract

Dipeptidyl peptidase-4 (DPP-4) inhibitors are reported to exhibit promising effects on several pathological processes associated with Parkinson’s disease (PD). To explore its repositioning potential as an antiparkinsonian agent, we evaluated the effects of omarigliptin (OMG), a DPP-4 inhibitor recently approved as a hypoglycemic drug, on neurotoxin-induced toxicity, using PC12 cells as a cellular model of PD. The molecular mechanism(s) underlying its protective activity was also investigated. OMG alleviated oxidative toxicity and the production of reactive oxygen species induced by 6-hydroxydopamine (6-OHDA) or rotenone. It also partially attenuated the formation of DPPH radicals and lipid peroxidation, demonstrating the antioxidant properties of OMG. OMG upregulated Nrf2 and heme oxygenase-1 (HO-1). Notably, treatment with a selective HO-1 inhibitor and Nrf2 knockdown by siRNA abolished the beneficial effects of OMG, indicating that the activated Nrf2/HO-1 signaling was responsible for the protective activity. Moreover, OMG exhibited anti-inflammatory activity, blocking inflammatory molecules, such as nitric oxide (NO) and inducible NO synthase, through inhibition of IκBα phosphorylation and NF-κB activation in an Akt-dependent fashion. Finally, OMG decreased the levels of cleaved caspase-3 and Bax and increased the level of Bcl-2, indicating its anti-apoptotic properties. Collectively, these results demonstrate that OMG alleviates the neurotoxin-induced oxidative toxicity through Nrf2/HO-1-mediated antioxidant, NF-κB-mediated anti-inflammatory, and anti-apoptotic mechanisms in PC12 cells. Our findings elucidating multiple mechanisms of antiparkinsonian activity strongly support the therapeutic potential of OMG in the treatment of PD.

## 1. Introduction

Parkinson’s disease (PD) is a progressive disorder characterized by both motor and non-motor complications [1]. To date, PD remains cureless; the current therapies just halt the motor symptoms without addressing the etiology of the disease. Thus, a therapeutic strategy targeting the core causes of the disease is still a clinically urgent necessity. Among various strategies to develop novel therapeutic interventions for PD, drug repositioning (also known as drug repurposing) has become an attractive approach to identifying potential candidates from the existing drugs that have been approved for medical applications [2,3]. This strategy is particularly appealing because it can save enormous amounts of the time and cost required for new drug development by traditional de novo processes. Moreover, the availability of pharmacological and safety profiles of the given drugs allows the acceleration of drug discovery with minimal risks [4,5,6].

Glucagon-like peptide-1 (GLP-1) is an endogenous hormone secreted by intestinal enteroendocrine cells in the gastrointestinal tract and the pancreas. The interaction of GLP-1 with its receptor (GLP-1R) is known to decrease blood glucose levels by boosting insulin secretion. For this reason, GLP-1R agonists, such as exenatide and liraglutide, are effectively used to treat type 2 diabetes mellitus (T2DM). Interestingly, the expression of GLP-1R has been found not only in the pancreas but also in the brain [7]. In the brain, insulin signaling is reported to play a key role in neuronal metabolism and repair as well as in synaptic plasticity [7]. The desensitized insulin signaling has been detected in the brain of patients with PD [7]. Neurons are particularly vulnerable to insulin resistance given that impaired insulin signaling exposes neurons to increased metabolic stress and augments neuronal dysfunction. However, the probable molecular basis for the deleterious involvement of insulin desensitization in the neurodegeneration of dopaminergic neurons in PD remains uncertain [8]. GLP-1 is neurotrophic, stimulating neurite outgrowth and preventing neurodegeneration in different cellular and animal models of various neurological disorders [9,10,11]. Therefore, GLP-1 signaling has been an attractive target for various neurodegenerative diseases, including PD.

**Figure 1:**
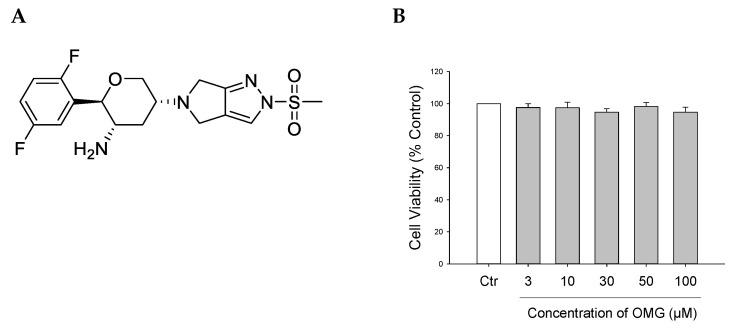
(**A**) Chemical structure of OMG. (**B**) Effect of OMG on the viability of PC12 cells. PC12 cells were treated with OMG at the indicated concentrations in media containing low serum (1%) for 24 h. MTT assays were performed as described in the Materials and Methods section. Data are expressed as the mean ± SEM of three independent experiments. OMG, omarigliptin.

GLP-1 has been reported to reverse the effects of neurotoxic compounds and pathological abnormalities associated with PD [12]. However, one major imperfection of GLP-1 is its short half-life, due to its rapid degradation by the enzyme dipeptidyl peptidase-4 (DPP-4). This gave rise to the development of DPP-4 inhibitors (gliptins), a relatively new class of oral glucose-lowering drugs that lead to the elevation of GLP-1 levels. Various DPP-4 inhibitors have shown promising effects in preclinical and clinical studies on several pathological processes of PD and Alzheimer’s disease (AD) [13,14,15,16,17]. Previous studies have shown that many DPP-4 inhibitors, such as sitagliptin [18,19,20], vildagliptin [15,21], saxagliptin, and linagliptin [22], exhibited antioxidant, anti-apoptotic, and neurorestorative properties [23]. Moreover, a recent study proposed repositioning teneligliptin and trelagliptin for treating brain disorders [24]. Omarigliptin (OMG, Figure 1A), a novel long-acting DPP-4 inhibitor recently approved for the management of T2DM [25], is the only gliptin possessing the ability to cross the blood–brain barrier (BBB) due to its low molecular weight and adequate lipophilic properties [26]. In addition, OMG has the advantage of oral administration once-weekly, thus enhancing patients’ compliance [7,26,27]. Furthermore, it was reported to alleviate cognitive dysfunction in streptozotocin-induced diabetic mice [28], and its combination with a dietary flavonoid, galangin, mitigated lipopolysaccharide-induced neuroinflammation in rats [29].

These findings prompted us to investigate the effects of OMG on neurotoxin-induced cell damage to endorse the possibility of its repositioning as an antiparkinsonian agent. As a cellular model of PD in this study, we used the 6-hydroxydopamine (6-OHDA)- or rotenone-treated rat pheochromocytoma (PC12) cell line. This cell-based model has been widely used to study the regulation of neuronal differentiation, neurosecretion, and biochemical and pathophysiological properties associated with neurodegenerative disorders, such as PD and AD [30,31,32]. Moreover, this cell line has been previously shown to express GLP-1R and used as a cell model of AD and PD to study the protective roles of GLP-1 and its analogs [11,33,34], validating our experimental system. In the current study, we first evaluated the protective effects of OMG against oxidative damage induced by 6-OHDA or rotenone in PC12 cells. In addition, we examined the antioxidant, anti-inflammatory, and anti-apoptotic properties of OMG. The signaling molecules mediating the effects of OMG were also elucidated in this study.

## 2. Materials and Methods

### 2.1. Materials

PC12 cells (CRL-1721) were obtained from the American Type Culture Collection (Manassas, VA, USA). Fetal bovine serum (FBS), horse serum (HS), antibiotic–antimycotic agents, and RPMI 1640 medium were bought from Gibco BRL (Grand Island, NY, USA). Zn(II)-protoporphyrin IX (ZnPP) and 6-OHDA were purchased from Tocris Bioscience (Bristol, UK). Dimethyl sulfoxide (DMSO), 3-(4,5-dimethyldiazol-2-yl)-2,5-diphenyltetrazolium bromide (MTT), 2′,7′-dichlorofluorescein diacetate (DCFH-DA), laminin, rotenone, 1,1-diphenyl-2-picrylhydrazyl (DPPH), trichloroacetic acid (TCA), 2-thiobarbituric acid (TBA), rabbit anti-lamin-B1 (1:2000, cat# SAB1306342), and mouse anti-β-actin (1:2000, cat#A5316) antibodies were purchased from Sigma-Aldrich (St. Louis, MO, USA). LY294002, antibodies specifically recognizing phosphorylated Akt (1;1000, cat#4058), inducible nitric oxide synthase (iNOS) (1:1000, cat# 2982), nuclear factor-kappa B (NF-κB) p65 (1:1000, cat# 6956), inhibitor of NF-κBα (IκBα) (1:1000, cat# 4814), B-cell lymphoma 2-associated X (Bax) (1:1000, cat# 2772), B-cell lymphoma 2 (Bcl-2) (1:2000, cat# 7382), and cleaved caspase-3 (1:1000, cat# 9661) were acquired along with horseradish peroxidase (HRP)-conjugated rabbit (1:2000, cat#7074) and mouse immunoglobulin G (IgG) (1:2000, cat#7076) from Cell Signaling Technology (Danvers, MA, USA). Anti-heme oxygenase-1 (HO-1) antibody (1:1000, cat#ADI-SPA-895-J) was procured from Enzo Life Sciences, Inc. (Farmingdale, NY, USA), and anti-nuclear factor erythroid 2-related factor 2 (Nrf2) antibody (1:1000, cat#16396-1) was bought from Proteintech (Pennsylvania, PA, USA). Anti-lamin-B1 antibody was provided by Abcam (Cambridge, MA, USA). SP600125 was supplied by Calbiochem (Darmstadt, Germany). Alexa Fluor 488-conjugated anti-mouse IgG and 4′,6-diamidino-2-phenylindole dihydrochloride (DAPI) were obtained from Thermo Scientific (Rockford, IL, USA). All the other chemicals were of analytical grade.

### 2.2. Cell Culture

PC12 cells were maintained in RPMI-1640 media containing 100 IU/mL penicillin, 100 μg/mL streptomycin, 10% HS, and 5% FBS in a humidified incubator, as previously described [30]. The culture media were changed twice weekly. Cells were plated onto 24-well plates pre-coated with poly-D-lysine/laminin at a density of 3 × 10^5^ cells/well and incubated for 24 h to induce cell adhesion.

### 2.3. Treatment of Cells

To estimate whether OMG exerted any cytotoxicity, PC12 cells were treated with OMG at a series of concentrations in culture media containing low serum (1% FBS, 1% HS, 100 IU/mL penicillin, and 100 μg/mL streptomycin) for 24 h. Control cells were treated with the same media containing low serum and an equivalent volume of DMSO (0.5%). The protective effects of OMG against 6-OHDA- or rotenone-induced toxicity in PC12 cells were evaluated as reported previously [30,35]. Briefly, cells were pretreated with OMG at various concentrations for 4 h and subsequently exposed to 6-OHDA (50 μM) or rotenone (1 μM) for 24 h. The effect of ZnPP, an HO-1 inhibitor, on the protective effects of OMG against 6-OHDA- or rotenone-induced toxicity was evaluated as reported [30]. In brief, the cells were pretreated with 0.2 µM ZnPP for 30 min and exposed to OMG for 4 h. Then, 6-OHDA or rotenone was added at the final concentrations of 50 and 1 µM, respectively, and incubated for 24 h.

### 2.4. Measurement of Cell Viability

After the desired treatment, cell viability was measured using the MTT assay as described [30,35]. Briefly, 0.5 mg/mL MTT was added to the cells and incubated at 37 °C for 3 h. The supernatants were cautiously taken out, and 300 μL DMSO was added to each well to liquefy the formazan precipitate. The absorbance of the liquefied solution was determined at 550 nm using a microplate reader (SpectraMax M2e, Molecular Devices, Sunnyvale, CA, USA). The cell viability was expressed as the percentage of the absorbance determined in the vehicle-treated control cells.

### 2.5. Measurement of Intracellular Reactive Oxygen Species (ROS) Levels

Intracellular ROS levels were measured using the fluorescent dye DCFH-DA, as reported previously, with slight modifications [36,37]. Briefly, cells were pretreated with OMG at various concentrations for 4 h and subsequently exposed to 50 μM 6-OHDA or 1 μM rotenone for 6 h. The duration of exposure to 6-OHDA or rotenone was determined from our preliminary time course study, exhibiting the maximum levels of ROS generation after 6 h of exposure in PC12 cells. The cells were then dyed with 25 μM DCFH-DA at 37 °C for 1 h. After washing the cells three times, the fluorescence was measured spectrophotometrically at an excitation wavelength of 490 nm and emission wavelength of 520 nm in a microplate reader (SpectraMax M2e, Molecular Devices). The ROS levels were expressed as the percentage of the vehicle-treated control cells. The fluorescent signals were also captured using a fluorescence microscope (Nikon Instruments, Inc., Melville, NY, USA).

### 2.6. Measurement of DPPH Radical Scavenging Activity

The DPPH radical scavenging activity of OMG was assessed by the cell-free bioassay, as previously described [37]. Briefly, the reaction mixture containing DPPH solution (150 µM prepared in methanol) and the desired concentrations of OMG was incubated at 37 °C for 30 min. The absorbance of the mixture was determined at 520 nm in a microplate reader (SpectraMax M2e, Molecular Devices). The DPPH radical scavenging activity was calculated using the following equation:

DPPH radical scavenging activity (%) = 100 × (Abs_control_ − Abs_sample_)/Abs_control_ (1)

where Abs_control_ and Abs_sample_ are the absorbances in the absence and presence of OMG, respectively.

### 2.7. Measurement of Lipid Peroxidation (LPO) in Rat Forebrain Homogenates

The effect of OMG on LPO initiated by Fe^2+^ and L-ascorbic acid in rat forebrain homogenates was evaluated as previously described [30,38,39]. Sprague–Dawley (SD) rats were supplied by Daehan Biolink (Chungbuk, Korea) and maintained at appropriate humidity (40–60%) and temperature (22 ± 2 °C) conditions in the animal facility. All the experimental protocols of the animal experiments were approved by the Institutional Animal Ethical Committee of Dongguk University (approval no.: IACUC-2019-001-2). In brief, an aliquot of SD rat forebrain homogenate (3–4 mg protein/mL) in 20 mM Tris-HCl buffer (pH 7.4) was added along with various concentrations of OMG to a reaction mixture containing Fe^2+^ (10 μM) and L-ascorbic acid (100 μM), and incubated at 37 °C for 1 h. The reaction was stopped by the sequential addition of 28% (*w*/*v*) TCA and 1% (*w*/*v*) TBA. Then, the mixture was boiled at 100 °C for 15 min and centrifuged at 4 °C at 3000 rpm for 10 min. The absorbance of the supernatant was determined at 532 nm in a microplate reader (SpectraMax M2e, Molecular Devices). The percentage of inhibition was calculated using the following equation:

Inhibition of LPO (%) = 100 × (1 − Abs_sample_/Abs_control_) (2)

where Abs_control_ and Abs_sample_ are the absorbances in the absence and presence of OMG, respectively.

### 2.8. Measurement of Nitric Oxide (NO) Levels

To evaluate the anti-inflammatory effect of OMG, NO levels were measured by the Griess reaction, as reported previously [40,41]. Overall, cells seeded onto 24-well plates at a density of 2 × 10^6^ cells/well were treated with OMG at various concentrations for 4 h and subsequently exposed to 6-OHDA (50 µM) or rotenone (1 µM) for an additional 24 h. After treatment, the media were carefully collected and centrifuged at 4 °C at 1500 rpm for 10 min. The supernatants were then stored at −80 °C until use. The concentrations of NO were determined using the standard curve generated simultaneously.

### 2.9. Western Blotting

PC12 cells were plated on 35 mm dishes at a density of 3 × 10^6^ cells/dish and maintained in a humidified incubator for 24 h. Cells were treated with the desired concentrations of OMG for the indicated periods of time. Control cells were treated with media containing low serum (1%) and 0.5% DMSO. Total cell lysates were acquired according to the procedures reported previously [30] and stored at −20 °C until use. To assess the effect of OMG on the nuclear translocation of Nrf2, the nuclear fraction was prepared from the cytosolic fraction using NE-PER Nuclear and Cytoplasmic Extraction Reagents (Thermo Scientific), according to the manufacturer’s instructions and as reported previously [41,42]. The protein concentrations were calculated using a Bio-Rad DC Protein Assay Kit (Bio-Rad, Hercules, CA, USA). Equal quantities of proteins (30 µg) were resolved by sodium dodecyl sulfate-polyacrylamide gel electrophoresis and electrophoretically transferred onto the nitrocellulose membrane (Whatman, Clifton, NJ, USA). Subsequently, immunoblotting was accomplished using specific antibodies recognizing the target proteins. Beta-actin was used as a loading control to standardize the level of cytosolic proteins, while lamin-B was used to standardize the level of nuclear proteins. The immunoreactive bands corresponding to the detected proteins were imagined using the Clarity™ Western ECL substrate (Bio-Rad) with the Bio-Rad ChemiDoc XRS imaging system (Bio-Rad).

### 2.10. Transitory Transfection with Small Interfering RNA (siRNA)

Control siRNA (siCtr) and siRNA targeting Nrf2 (siNrf2, cat# sc-156128) were purchased from Santa Cruz Biotechnology, Inc. (Dallas, TX, USA). PC12 cells were transfected with siCtr or siNrf2 using the Lipofectamine 2000 transfection reagent and Opti-MEM I reduced serum medium (Thermo Scientific), as reported previously [42,43]. Briefly, cells were incubated in media without antibiotic–antimycotic until reaching 70–90% confluency. Opti-MEM I and siRNA were mixed to make solution A, and Opti-MEM I and Lipofectamine 2000 were mixed to make solution B. The two solutions were then left in situ for 5 min and gently mixed to yield solution C. After 20 min, the culture media were replaced with fresh media without antibiotics containing solution C at a final concentration of 100 nM siRNA. After 6 h of transfection, the media containing siRNA were exchanged with fresh media containing antibiotic–antimycotic. After 36–48 h with daily media exchanges, the cells were used for further experiments.

### 2.11. Immunocytochemistry

To assess the effect of OMG on the nuclear translocation of NF-κB, immunocytochemistry was performed according to the procedures reported previously [41,42]. In brief, cells were plated on coverslips placed on the wells of 24-well plates at a density of 1 × 10^6^ cells/well. After incubation for 24 h, the cells were treated with OMG (30 and 50 μM) in media containing 1% serum. Then, the cells were fixed with 4% paraformaldehyde for 15 min and permeabilized with 0.3% Triton X-100 for 15 min prior to blocking with 5% goat serum for 30 min. The cells were incubated with the anti-NF-κB antibody (1:250 dilution) in the blocking solution at 4 °C overnight and exposed to Alexa Fluor 488-conjugated secondary antibody (1:400 dilution) at room temperature for 1 h in the dark. The coverslips were gently washed with phosphate-buffered saline and mounted with ProLong Gold Antifade Reagent with DAPI onto microscope slides. The final samples were examined using a confocal microscope (Nikon Instruments, Inc.).

### 2.12. Statistical Analysis

All data are displayed as the mean ± SEM of at least three independent experiments. Statistical significances were analyzed by one-way analysis of variance (ANOVA) with SigmaPlot 12.5 software (Systat Software, Inc., San Jose, CA, USA). A *p* < 0.05 was considered statistically significant.

## 3. Results and Discussion

### 3.1. Effect of OMG on Viability of PC12 Cells

Prior to evaluating the potential protective effects of OMG against neurotoxin-induced cell death, its cytotoxicity profile was assessed in PC12 cells. The cells were treated with OMG at 3, 10, 30, 50, and 100 μM for 24 h, and cell viability was assessed by MTT assay. We found that OMG did not significantly alter cell viability at the concentrations tested (Figure 1B). Accordingly, further experiments were carried out with OMG at concentrations below 100 μM.

### 3.2. Effect of OMG on 6-OHDA- or Rotenone-Induced Toxicity in PC12 Cells

**Figure 2:**
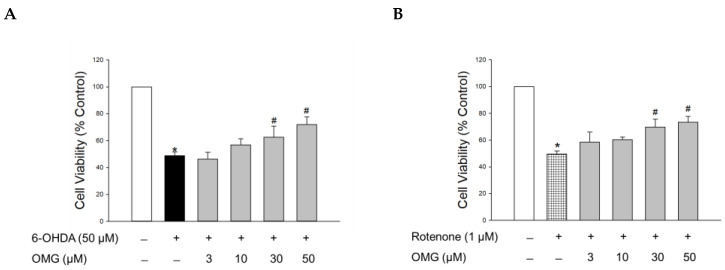
Effects of OMG on 6-OHDA- or rotenone-induced oxidative toxicity in PC12 cells. PC12 cells were pretreated with OMG at the indicated concentrations for 4 h and subsequently exposed to (**A**) 6-OHDA or (**B**) rotenone for an additional 24 h. Cell viability was evaluated by MTT assay, as described in the Materials and Methods section. Data are displayed as the mean ± SEM of three independent experiments. *, *p* < 0.05 and ^#^, *p* < 0.05 vs. vehicle-treated control cells and 6-OHDA- or rotenone-treated cells, respectively. OMG, omarigliptin; 6-OHDA, 6-hydroxydopamine.

The hydroxylated derivative of dopamine, 6-OHDA, is widely used to induce cell-based and in vivo experimental models of PD. Following cellular uptake, 6-OHDA induces dopaminergic neurotoxicity by generating intracellular ROS, resulting in oxidative cell death [44]. It also causes mitochondrial dysfunction, ultimately leading to apoptosis [35,44]. Accordingly, we assessed the protective capability of OMG against the toxicity induced by 6-OHDA in PC12 cells. To evaluate its effect on the 6-OHDA-induced oxidative toxicity, cells were pretreated with serial concentrations of OMG (3, 10, 30, and 50 μM) for 4 h and exposed to 6-OHDA (50 μM) for an additional 24 h. Cell viability was then analyzed by MTT assay. Consistent with previous reports [30,35,45], 6-OHDA treatment remarkably reduced the cell viability to 48.7 ± 2.7% of the control (Figure 2A). Pretreatment with OMG presented a significant reversal of the 6-OHDA-induced toxicity at concentrations of 30 and 50 μM.

We further evaluated the effect of OMG on the toxicity induced by rotenone. Rotenone, a well-known lipophilic neurotoxin that can penetrate the BBB, inhibits complex I of the mitochondrial respiratory chain in dopaminergic neurons and induces mitochondrial membrane depolarization, ultimately resulting in neuronal toxicity. This process is also associated with oxidative stress mediated by abnormally enhanced levels of ROS and NO [46]. Hence, rotenone-treated PC12 cells are also widely used as a cell model of PD, mimicking its pathophysiology [46,47,48,49,50]. As reported previously [30,35], the cells exposed to rotenone (1 μM) showed about 50% viability relative to the vehicle-treated control cells. Similar to 6-OHDA-induced toxicity, rotenone-induced toxicity was also markedly inhibited by OMG at concentrations of 30 and 50 μM (Figure 2B). These results demonstrated that OMG attenuated 6-OHDA- or rotenone-induced cytotoxicity in PC12 cells.

### 3.3. Effect of OMG on 6-OHDA- or Rotenone-Induced ROS Generation in PC12 Cells

**Figure 3:**
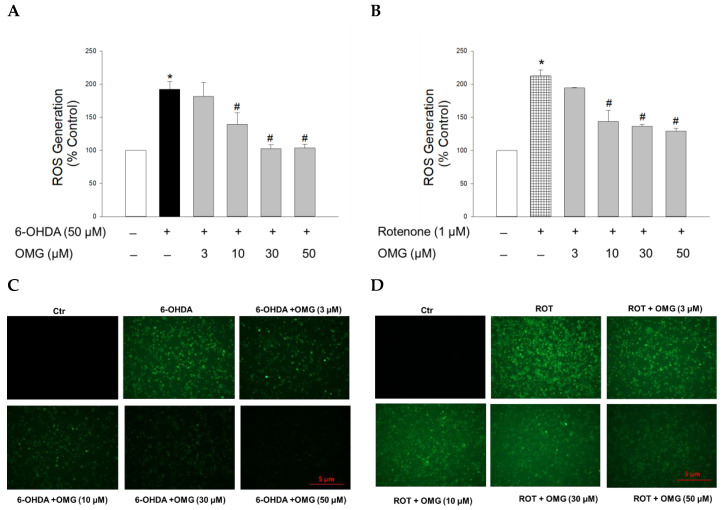
Effects of OMG on the 6-OHDA- or rotenone-induced ROS generation in PC12 cells. PC12 cells were pretreated with OMG at the indicated concentrations for 4 h and subsequently exposed to (**A**) 6-OHDA or (**B**) rotenone for 6 h. Then, cells were loaded with 25 μM DCFH-DA at 37 °C for 1 h in the dark, and the intracellular ROS levels were assessed by the fluorescence emission of 2′,7′-dichlorofluorescein, as described in the Materials and Methods section. Intracellular levels of ROS are presented as the percentage of the vehicle-treated control cells. Each data point is displayed as the mean ± SEM from three independent experiments conducted in triplicate. *, *p* < 0.05 and ^#^, *p* < 0.05 vs. vehicle-treated control cells and 6-OHDA- or rotenone-treated cells, respectively. Immunofluorescence images of the cells treated with OMG and (**C**) 6-OHDA or (**D**) rotenone were obtained as described in the Materials and Methods. Representative images are shown (scale bar, 5 μm). OMG, omarigliptin; 6-OHDA, 6-hydroxydopamine; ROT, rotenone; ROS, reactive oxygen species; Ctr, control.

It is believed that oxidative stress is a crucial precursor for triggering injury to dopaminergic neurons in PD [35,39]. As described above, both 6-OHDA and rotenone are known to induce oxidative stress through upregulation of intracellular ROS levels [44,46]. Thus, we next examined whether OMG affected the ROS generation induced by 6-OHDA or rotenone in PC12 cells after staining the cells with fluorescent dye DCFH-DA. Intracellular ROS levels in PC12 cells treated with 6-OHDA (50 µM) or rotenone (1 µM) for 6 h were increased approximately two-fold compared with control cells (Figure 3). OMG substantially reduced the ROS production induced by 6-OHDA (Figure 3A) or rotenone (Figure 3B) in a concentration-dependent manner. The inhibition of ROS generation by OMG in 6-OHDA- or rotenone-treated PC12 cells was further validated by the fluorescence microscopy images, displaying a marked reduction in the intensity of 2′,7′-dichlorofluorescein fluorescence in the presence of OMG (Figure 3C,D). These findings demonstrate the antioxidant properties of OMG, inhibiting the 6-OHDA- or rotenone-induced production of intracellular ROS in PC12 cells.

### 3.4. Effects of OMG on DPPH Radical Formation and LPO

**Figure 4:**
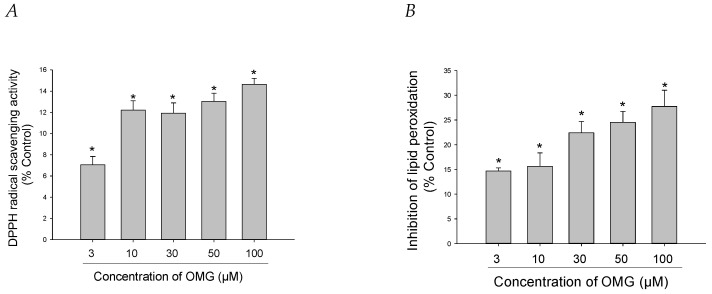
Effects of OMG on DPPH radical formation and lipid peroxidation (LPO). (**A**) DPPH radical formation and (**B**) LPO initiated by Fe^2+^ (10 µM) and L-ascorbic acid (100 µM) in rat forebrain homogenates were evaluated as described in the Materials and Methods section. Data are displayed as the mean ± SEM from three independent experiments conducted in duplicate. *, *p* < 0.05 vs. DPPH radicals and LPO measured in the absence of OMG, respectively. OMG, omarigliptin; DPPH, 1,1-diphenyl-2-picrylhydrazyl; LPO, lipid peroxidation.

The antioxidant capacities of OMG were further explored by assessing its ability to scavenge DPPH radicals in a cell-free assay. We found that OMG displayed minimal but significant DPPH radical scavenging activity, with the highest scavenging effect of approximately 14% at 100 µM (Figure 4A). We also examined the capability of OMG to inhibit LPO initiated by Fe^2+^ and L-ascorbic acid in rat forebrain homogenates. OMG reduced lipid peroxide formation by 27.7 ± 3.2% at 100 μM (Figure 4B). Collectively, our findings imply that inhibition of LPO by OMG and its radical scavenging activity contribute, at least in part, to its antioxidant activity, eliminating the intracellular ROS induced by 6-OHDA or rotenone in PC12 cells.

### 3.5. Effect of OMG on the Activation of Nrf2/HO-1 Signaling and Its Role in 6-OHDA- or Rotenone-Induced Toxicity in PC12 Cells

**Figure 5:**
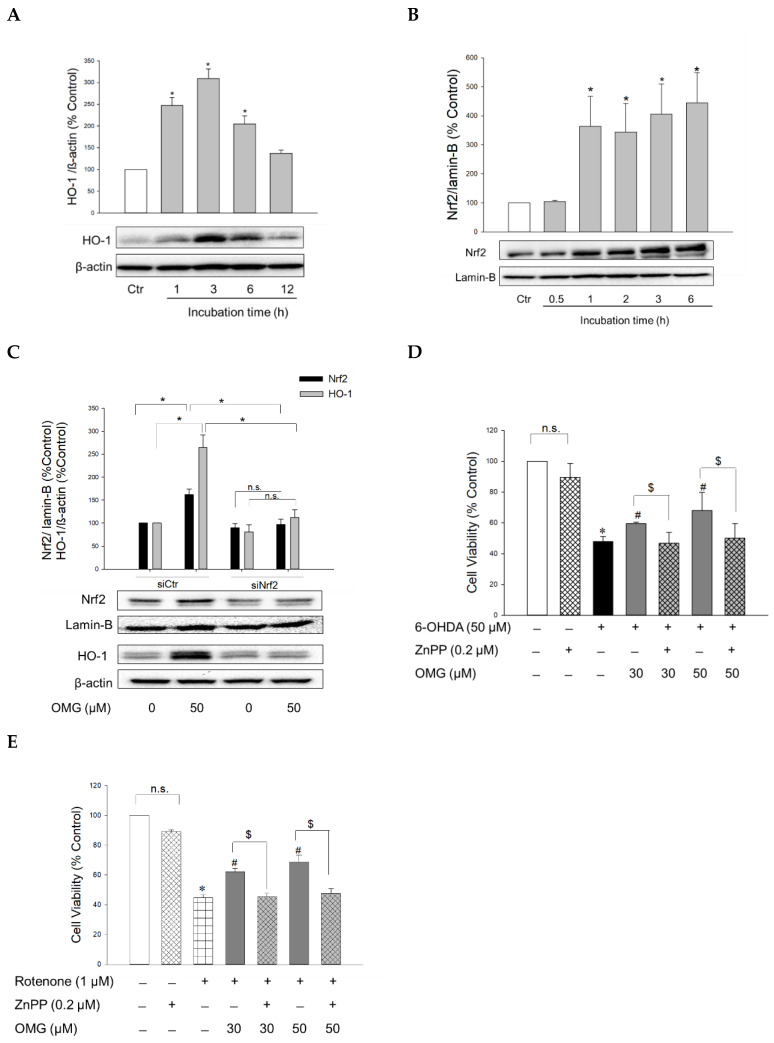
Activation of Nrf2/HO-1 signaling by OMG and its role in 6-OHDA- or rotenone-induced toxicity in PC12 cells. (**A**,**B**) PC12 cells were treated with OMG at 50 µM at the indicated time points, and the expression levels of (**A**) cytosolic HO-1 and (**B**) nuclear Nrf2 were evaluated by western blotting as described in the Materials and Methods section. Band intensities were quantified by densitometric analyses, and the cytosolic and nuclear proteins were normalized to β-actin and lamin-B, respectively. Data are displayed as the mean ± SEM from at least three independent experiments. *, *p* < 0.05 vs. vehicle-treated control cells. (**C**) PC12 cells were transfected with 100 nM of control siRNA (siCtr) or Nrf2-targeted siRNA (siNrf2) for 48 h and then treated with 50 μM OMG for an additional 6 h. Nuclear Nrf2 and cytosolic HO-1 levels were analyzed by western blotting, as described in the Materials and Methods. Band intensities were quantified by densitometric analyses, and the nuclear and cytosolic proteins were normalized to lamin-B and β-actin, respectively. (**D**,**E**) PC12 cells were pretreated with 0.2 µM ZnPP for 30 min and subsequently exposed to OMG (30 and 50 µM) for 4 h, followed by incubation with (**D**) 6-OHDA or (**E**) rotenone for an additional 24 h. Cell viability was evaluated by MTT assay. Data are displayed as the mean ± SEM from three measurements performed in duplicates. *, *p* < 0.05 vs. vehicle-treated control cells; ^#^, *p* < 0.05 vs. 6-OHDA- or rotenone-treated cells; ^$^, *p* < 0.05 vs. cells treated with OMG and 6-OHDA or rotenone without ZnPP. Ctr, control; HO-1, heme oxygenase-1; Nrf2, nuclear factor erythroid 2-related factor 2; OMG, omarigliptin; ZnPP, Zn(II)-protoporphyrin IX; 6-OHDA, 6-hydroxydopamine; n.s., not significant.

The transcription factor Nrf2 controls the expression of antioxidant defense enzymes in response to oxidative stress [51,52]. Nrf2 translocates to the nucleus, where it binds to antioxidant response elements (AREs), triggering the transcription of ARE genes, including the gene encoding HO-1. HO is an enzyme that catalyzes the degradation of heme to produce biliverdin, ferrous ion, and carbon monoxide. Unlike HO-2 which is a constitutive isoform, HO-1 is inducible. This isoform has been the subject of extensive study due to its cytoprotective benefits, that is, its antioxidative, anti-inflammatory, and anti-apoptotic properties [53,54]. To elucidate the molecular mechanism(s) underlying the protective effects of OMG against 6-OHDA- or rotenone-induced toxicity, the effect of OMG on HO-1 expression was examined in PC12 cells. The cells were treated with OMG at 50 μM for the indicated periods of time (1, 3, 6, and 12 h), and the levels of HO-1 expression were measured by western blotting. As shown in Figure 5A, OMG markedly augmented the expression of HO-1, with the maximal increase at 3 h of treatment. We then investigated whether OMG could enhance Nrf2 nuclear translocation. Western blotting analyses demonstrated that 50 μM OMG dramatically enhanced the nuclear translocation of Nrf2 up to four-fold, as early as after 1 h of incubation in PC12 cells (Figure 5B). The fact that Nrf2 reached its maximum translocation prior to HO-1 upregulation, suggested that Nrf2 could be the upstream signal of HO-1 induction. To validate this possibility, PC12 cells were transfected with siNrf2, and the levels of Nrf2 and HO-1 expression were determined in the presence or absence of OMG by western blotting. As shown in Figure 5C, siRNA-mediated Nrf2 knockdown completely suppressed OMG-induced HO-1 upregulation, demonstrating that Nrf2 activation is required for HO-1 induction by OMG in PC12 cells.

To reveal the role of Nrf2-mediated HO-1 induction in the protective effects of OMG, cells were pretreated with ZnPP, a selective HO-1 inhibitor, and subsequently exposed to 6-OHDA or rotenone in the presence or absence of OMG at 30 and 50 μM. Cell viability was then evaluated by MTT assay. We found that the protection by OMG against 6-OHDA- or rotenone-induced toxicity was fully abolished by ZnPP (Figure 5D,E). Cells treated with ZnPP and 6-OHDA or rotenone in the presence of OMG (30 and 50 μM) exhibited the same cell viability as the cells treated with 6-OHDA or rotenone without OMG. The viability of PC12 cells was unaffected by ZnPP alone (Figure 5D,E). Based on these findings, the upregulation of HO-1 expression by OMG plays a crucial role in counteracting 6-OHDA- or rotenone-induced toxicity in PC12 cells.

### 3.6. Effect of OMG on NO Production and iNOS Expression in 6-OHDA- or Rotenone-Treated PC12 Cells

GLP-1 signaling plays a key role in regulating neuroinflammation and memory function [55]. Neuroinflammatory processes have been associated with the pathogenesis of PD [56,57]. Moreover, evidence supports that controlling inflammatory processes can be exploited as promising interventional targets for PD and other neurodegenerative diseases, such as AD [58]. Therefore, we investigated the anti-inflammatory power of OMG in 6-OHDA- and rotenone-induced inflammatory processes.

**Figure 6:**
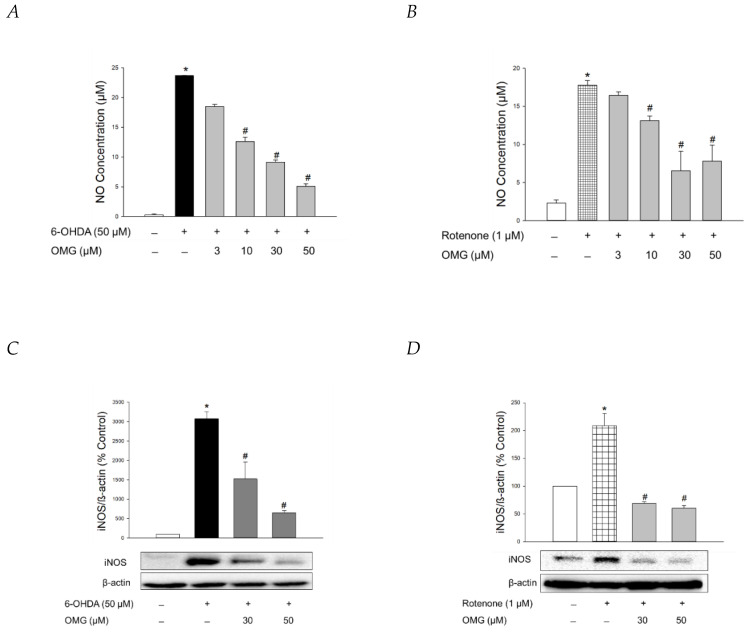
Inhibition of NO production and iNOS expression by OMG in 6-OHDA- or rotenone-treated PC12 cells. (**A**,**B**) PC12 cells were pretreated with OMG at the indicated concentrations for 4 h and subsequently exposed to (**A**) 6-OHDA or (**B**) rotenone for 24 h. Control cells were treated with the vehicle alone. NO production was evaluated by Griess assay, as described in the Materials and Methods section. NO concentrations were determined using the standard curve generated simultaneously. (**C**,**D**) Levels of iNOS expression were analyzed by western blotting, as described in the Materials and Methods. Band intensities were quantified by densitometric analyses and normalized to β-actin. Representative blots are shown. Data are displayed as the mean ± SEM from three independent experiments. *, *p* < 0.05 and ^#^, *p* < 0.05 vs. vehicle-treated control and 6-OHDA- or rotenone-treated cells, respectively. NO, nitric oxide; iNOS, inducible NO synthase; OMG, omarigliptin; 6-OHDA, 6-hydroxydopamine.

NO production is closely linked to the pathogenesis of neurodegenerative diseases, including PD [59,60]. Using Griess assays, we explored whether OMG exerted any effect on the production of NO induced by 6-OHDA or rotenone in PC12 cells. In parallel with previous findings [61,62,63], 6-OHDA or rotenone drastically enhanced the level of pro-inflammatory factor NO in PC12 cells (Figure 6A,B). OMG dramatically inhibited the 6-OHDA- and rotenone-induced NO production (Figure 6A,B).

The molecular mechanisms by which 6-OHDA destroys dopaminergic neurons remain elusive, but it has been proposed that the neurotoxin enters the cells via a dopamine transporter to subsequently promote chronic inflammatory processes and oxidative stress [64]. Rotenone has also been shown to trigger gene expression of pro-inflammatory proteins like iNOS, producing large quantities of NO during inflammatory processes [63]. Accordingly, we evaluated the effect of OMG on the 6-OHDA- and rotenone-induced expression of iNOS in PC12 cells. Western blotting analyses revealed that OMG at the concentrations of 30 and 50 μM markedly reversed the expression of iNOS in 6-OHDA- or rotenone-treated PC12 cells (Figure 6C,D).

### 3.7. Effect of OMG on 6-OHDA- or Rotenone-Induced Nuclear Translocation of NF-κB in PC12 Cells

**Figure 7:**
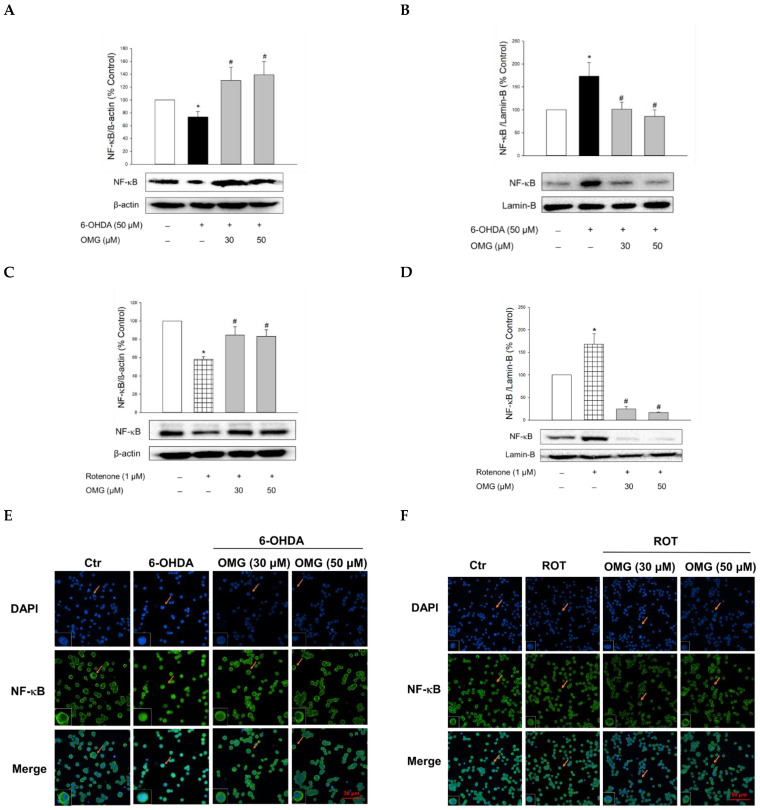
Inhibition of NF-κB nuclear translocation by OMG in 6-OHDA- or rotenone-treated PC12 cells. PC12 cells were pretreated with OMG at 30 and 50 μM for 4 h and subsequently exposed to 6-OHDA or rotenone for 6 h. (**A**–**D**) Cytosolic and nuclear levels of NF-κB were evaluated by western blotting using anti-NF-κB antibody, as described in the Materials and Methods section. Band intensity was quantified by densitometric analyses, and nuclear and cytosolic levels of NF-κB were normalized to lamin-B and β-actin, respectively. Data are displayed as the mean ± SEM from at least three independent experiments. *, *p* < 0.05 and ^#^, *p* < 0.05 vs. the vehicle-treated control and 6-OHDA- or rotenone-treated cells, respectively. (**E**,**F**) Immunocytochemical analysis was carried out using DAPI and anti-NF-κB antibodies, as described in the Materials and Methods. Representative images are shown. The arrow in each image indicates the magnified cell shown in the inset (scale bars in (**E**,**F**), 30 and 50 μm, respectively). NF-κB, nuclear factor-kappa B; OMG, omarigliptin; 6-OHDA, 6-hydroxydopamine; ROT, rotenone; Ctr, control; DAPI, 4′,6-diamidino-2-phenylindole dihydrochloride.

NF-κB is a key signaling molecule regulating the expression of inflammatory genes, such as iNOS. Upon its translocation to the nucleus, NF-κB induces the transcription of a series of genes involved in the inflammatory processes [65,66]. Thus, we examined the involvement of the nuclear translocation of NF-κB in the anti-inflammatory activities of OMG. Compared with the vehicle treatment, 6-OHDA or rotenone significantly increased the nuclear levels of NF-κB while the cytosolic levels of NF-κB decreased (Figure 7A–D), demonstrating the translocation of NF-κB to the nucleus. Treatment with OMG significantly inhibited the 6-OHDA- or rotenone-induced nuclear translocation of NF-κB. These findings were confirmed by immunocytochemical analysis (Figure 7E,F). The NF-κB p65 subunit was primarily localized in the cytoplasm in the vehicle-treated control cells but moved to the nucleus upon exposure to 6-OHDA or rotenone. Again, OMG at 30 and 50 µM suppressed the 6-OHDA- or rotenone-induced nuclear translocation of NF-κB (Figure 7E,F). Collectively, our findings indicate that OMG exerts anti-inflammatory effects by blocking NO production and iNOS expression through inhibition of NF-κB nuclear translocation in 6-OHDA- or rotenone-treated PC12 cells.

### 3.8. Effect of OMG on Phosphorylation of Akt and Its Role in NF-κB Signaling in PC12 Cells

Accumulating evidence supports that stimulation of GLP-1R in neurons leads to phosphorylation of Akt, which subsequently influences various downstream signaling pathways, such as NF-κB [58,67,68]. Accordingly, we explored whether OMG inhibited nuclear translocation of NF-κB by interfering with Akt phosphorylation in 6-OHDA- or rotenone-treated PC12 cells.

**Figure 8:**
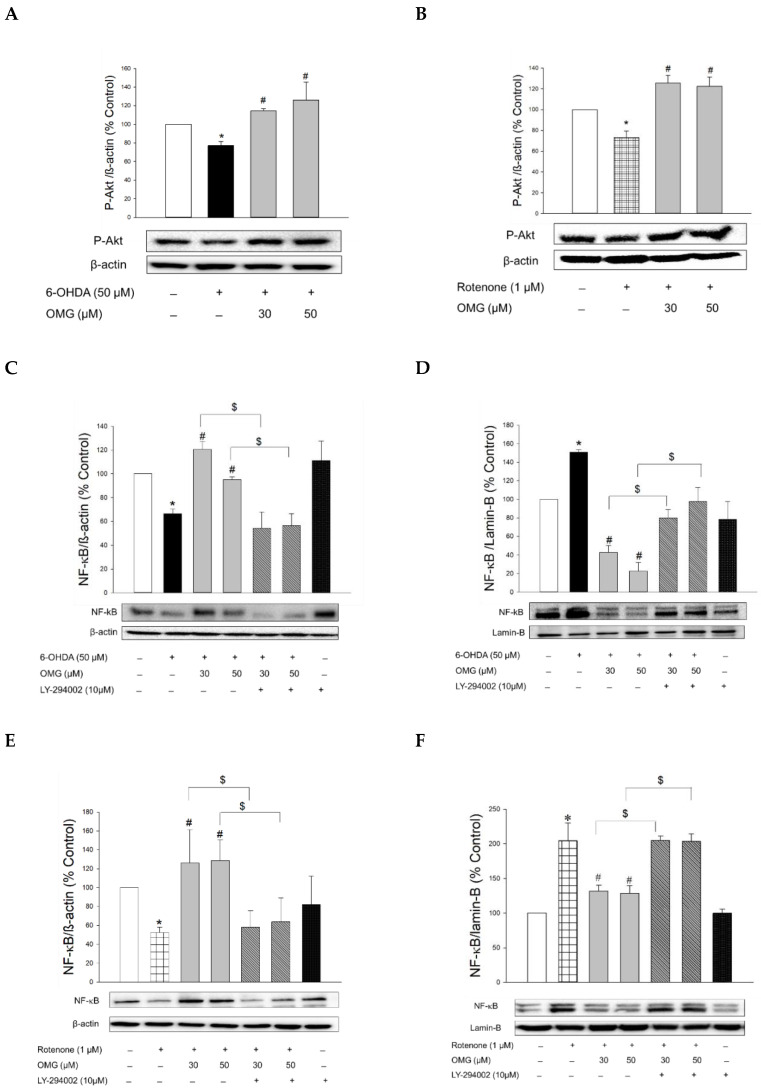

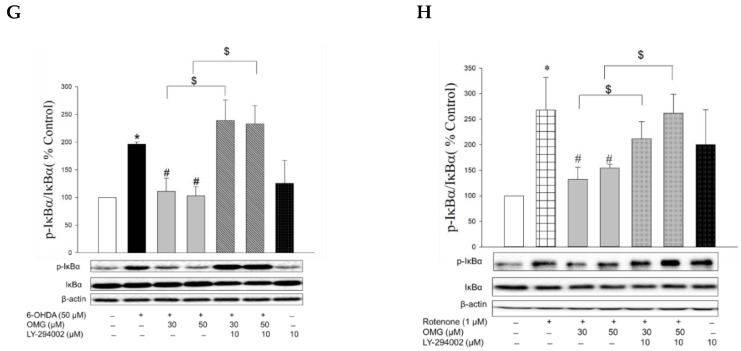
Induction of Akt phosphorylation by OMG and its role in the regulation of NF-κB signaling in PC12 cells. (**A**,**B**) PC12 cells were pretreated with OMG (30 and 50 μM) for 4 h, subsequently exposed to (**A**) 6-OHDA or (**B**) rotenone for 6 h, and the levels of phosphorylated Akt (p-Akt) were determined by western blotting using anti-p-Akt antibody, as described in the Materials and Methods section. (**C**–**H**) PC12 cells were pretreated with LY294002 (10 μM) for 2 h, followed by treatment with OMG (30 and 50 μM) for 4 h, and then exposed to (**C**,**D**,**G**) 6-OHDA or (**E**,**F**,**H**) rotenone for an additional 6 h. (**C**,**E**) Cytosolic and (**D**,**F**) nuclear levels of (**G**,**H**) NF-κB or phosphorylated IκBα (p-IκBα) were determined by western blotting using anti-NF-κB antibody and anti-p-IκBα or anti-IκBα antibodies, respectively, as described in the Materials and Methods. Band intensity was quantified by densitometric analyses. Cytosolic and nuclear levels of NF-κB were normalized to β-actin and lamin-B, respectively. P-IκBα levels were normalized to non-p-IκBα and β-actin. Data are displayed as the mean ± SEM from at least three independent experiments. *, *p* < 0.05 vs. the vehicle-treated control cells; ^#^, *p* < 0.05 vs. 6-OHDA- or rotenone-treated cells; ^$^, *p* < 0.05 vs. cells treated with 6-OHDA or rotenone and OMG. OMG, omarigliptin; NF-κB, nuclear factor-kappa B; 6-OHDA, 6-hydroxydopamine.

We first observed that the phosphorylation of Akt was downregulated in the cells treated with 6-OHDA or rotenone (Figure 8A,B). However, when the cells were pretreated with OMG at 30 or 50 μM, the downregulated p-Akt was significantly increased compared to the cells treated with 6-OHDA or rotenone alone, without OMG. To validate whether the phosphorylation of Akt by OMG is the upstream modulator of NF-κB, cells were pretreated with LY294002 (10 μM), a specific inhibitor of Akt, subsequently exposed to 6-OHDA in the presence or absence of OMG, and then the nuclear and cytosolic levels of NF-κB were determined by western blotting. As described earlier in this study (Figure 7A,B,E), OMG was a prominent inhibitor of nuclear translocation of NF-κB in 6-OHDA-treated cells (Figure 8C,D). Importantly, LY294002 dramatically reversed the inhibition of NF-κB translocation by OMG, demonstrating that Akt phosphorylation was essential for the inhibition of NF-κB translocation. Similarly, OMG inhibited the rotenone-induced NF-κB nuclear translocation, which was also reversed by LY294002 (Figure 8E,F).

The nuclear translocation of NF-κB is mediated by the phosphorylation of IκBα. Various inflammatory stimuli can trigger the phosphorylation of IκBα and thereby liberate NF-κB from the NF-κB–IκBα complex to allow its translocation to the nucleus [69]. Accordingly, we examined the effects of OMG on the phosphorylation of IκBα in the 6-OHDA- or rotenone-treated cells. We found that 6-OHDA or rotenone increased the phosphorylation of IκBα, and the increased IκBα phosphorylation was significantly inhibited by OMG (Figure 8G,H). Again, LY294002 dramatically restored the IκBα phosphorylation to levels similar to those observed in the cells treated with 6-OHDA or rotenone alone (Figure 8G,H), demonstrating the role of Akt in IκBα phosphorylation.

It has been reported that glycogen synthase kinase-3β (GSK-3β) participates in the regulation of NF-κB through the phosphorylation of IκBα [70,71]. GSK-3β is constitutively active in quiescent cells, and its activity can be regulated by several mechanisms. The most well-defined regulatory mechanism is the inhibition of its activity through phosphorylation at Ser9 by the phosphatidylinositol 3-kinase/Akt signaling pathway [72]. In the current study, we found that the decreased IκBα phosphorylation by OMG occurred in an Akt-dependent manner (Figure 8G,H). Given these findings, it would be valuable to further examine whether the Akt activated by OMG could trigger phosphorylation of GSK-3β at Ser9 and thereby inhibit IκBα phosphorylation in 6-OHDA- or rotenone-treated PC12 cells. This possibility is under investigation, along with analyses to reveal other signaling molecules involved in the inhibition of NF-κB activity by OMG. Future research is also required to determine the mechanisms by which OMG induces Akt phosphorylation.

Collectively, we demonstrated that OMG induced Akt phosphorylation and inhibited the 6-OHDA- or rotenone-induced IκBα phosphorylation and NF-κB translocation in PC12 cells. The decreased IκBα phosphorylation and NF-κB translocation by OMG were reliably reversed by LY294002, indicating the critical role of Akt in the regulation of NF-κB. These findings provide a plausible signaling pathway, which links the activation of GLP-1R through inhibition of DPP-4 by OMG and its anti-inflammatory effect of inhibiting NO production and iNOS expression in 6-OHDA- or rotenone-treated PC12 cells (Figure 6). It would be worth excluding the possibility that the neurotoxins used in this study directly altered GLP-1R expression and its signaling in PC12 cells.

### 3.9. Effects of OMG on Expression of Apoptosis-Related Proteins in 6-OHDA- or Rotenone-Treated PC12 Cells

**Figure 9:**
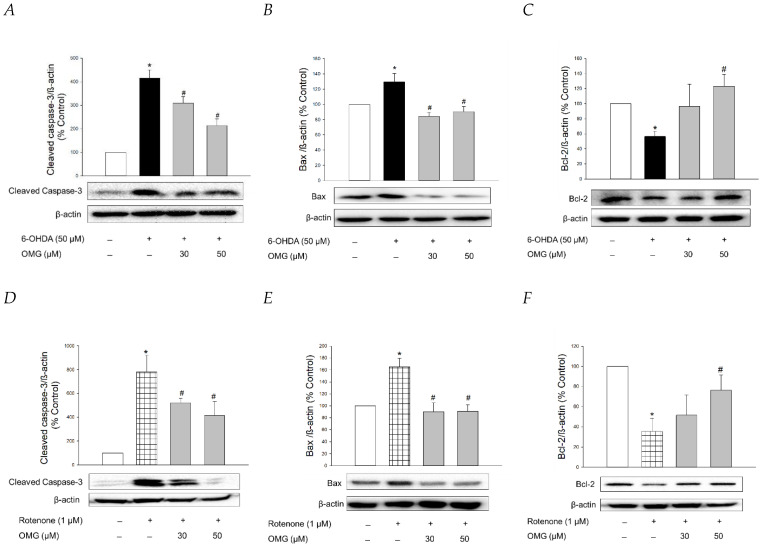
Anti-apoptotic properties of OMG in 6-OHDA- or rotenone-treated PC12 cells. PC12 cells were pretreated with OMG (30 and 50 μM) for 4 h, subsequently exposed to (**A**–**C**) 6-OHDA or (**D**–**F**) rotenone for an additional 6 h, and the expression levels of (**A**,**D**) cleaved caspase-3, (**B**,**E**) Bax, and (**C**,**F**) Bcl-2 were detected by western blotting, as described in the Materials and Methods sections. Band intensity was quantified by densitometric analyses. β-Actin was used for the normalization of each protein. Representative blots are shown. Data are displayed as the mean ± SEM from at least three independent experiments. *, *p* < 0.05 and ^#^, *p* < 0.05 vs. the vehicle-treated control and 6-OHDA- or rotenone-treated cells, respectively. OMG, omarigliptin; 6-OHDA, 6-hydroxydopamine; Bcl-2, B-cell lymphoma 2; Bax, Bcl-2-associated X.

It is well-established that apoptosis accompanied by mitochondrial dysfunction and oxidative stress is involved in 6-OHDA- or rotenone-induced cytotoxicity in PC12 cells [73,74,75]. To clarify whether OMG exhibited anti-apoptotic properties in PC12 cells, we studied the effect of OMG on the expression of typical apoptosis-related proteins, such as cleaved caspase-3, Bax, and Bcl-2, in 6-OHDA- or rotenone-treated PC12 cells by western blotting. Consistent with the previous reports [45,68], the levels of cleaved caspase-3 and Bax, well-known pro-apoptotic proteins, were significantly enhanced by 6-OHDA (Figure 9A,B). By contrast, the expression of Bcl-2, an anti-apoptotic protein, was remarkably decreased in 6-OHDA-treated cells (Figure 9C). The increased levels of cleaved caspase-3 and Bax and the decreased Bcl-2 expression indicated that 6-OHDA induced apoptotic cell death in PC12 cells. OMG explicitly reversed the increased levels of cleaved caspase-3 and Bax at both 30 and 50 µM, while it significantly enhanced Bcl-2 expression to the control level at 50 µM (Figure 9A–C). Rotenone also induced enhancement of cleaved caspase-3 and Bax (Figure 9D,E) and decreased Bcl-2 expression (Figure 9F). As seen in the 6-OHDA-treated cells, these apoptotic characteristics induced by rotenone were abolished by OMG (Figure 9D–F). These results verified that OMG exhibits anti-apoptotic activity in 6-OHDA- or rotenone-treated PC12 cells by inhibiting the expression of pro-apoptotic proteins and enhancing an anti-apoptotic molecule. In addition to its antioxidant and anti-inflammatory activity, the anti-apoptotic properties of OMG may also contribute to its protective effects against 6-OHDA- or rotenone-induced toxicity in PC12 cells. It would be interesting to further explore the effect of OMG on the other signaling molecules involved in the apoptotic process.

## 4. Conclusions

DPP-4 inhibitors (gliptins) are a relatively new class of oral hypoglycemic drugs to reduce blood glucose by increasing GLP-1 levels. Various DPP-4 inhibitors have been shown to exhibit promising effects in several preclinical and clinical studies for the treatment of neurodegenerative disorders, including PD and AD. OMG is a novel DPP-4 inhibitor recently approved for the management of T2DM with the advantages of a long half-life and BBB penetrability. To explore its repositioning potential as an antiparkinsonian agent, the present study evaluated the effects of OMG on the toxicity induced by 6-OHDA or rotenone in PC12 cells and elucidated the molecular mechanisms underlying its effects.

We found that OMG markedly protected PC12 cells against the toxicity of 6-OHDA or rotenone. Its antioxidant properties also resulted in the inhibition of 6-OHDA- or rotenone-induced production of ROS and partial attenuation of the formation of DPPH radicals and LPO in rat brain homogenates. Furthermore, OMG augmented the expression of the antioxidant enzyme HO-1 and increased the level of nuclear Nrf2, the transcription factor regulating the expression of antioxidant proteins. The protective effects of OMG were eliminated by ZnPP, an HO-1 inhibitor, demonstrating the critical role of HO-1 in its protective activity. Moreover, the upregulated HO-1 by OMG was abolished by Nrf2 knockdown, verifying that Nrf2 was the upstream regulator of HO-1. These findings demonstrated that OMG exhibited protective and antioxidant activity through the activation of the Nrf2/HO-1 signaling pathway.

In addition to its antioxidant activity, the anti-inflammatory activity of OMG was also established in this study. OMG decreased the 6-OHDA- and rotenone-induced IκBα phosphorylation and nuclear translocation of NF-κB, resulting in reduced production of the inflammatory mediator NO and iNOS expression. Notably, the inhibition of IκBα phosphorylation and NF-κB translocation by OMG was completely abolished by the Akt inhibitor (LY294002), indicating that the activation of Akt was the upstream signaling molecule regulating NF-κB activity in 6-OHDA- or rotenone-treated cells. Finally, the anti-apoptotic function of OMG was identified. OMG reduced the levels of cleaved caspase-3 and Bax in 6-OHDA- or rotenone-treated PC12 cells, while it increased Bcl-2 expression.

**Figure 10:**
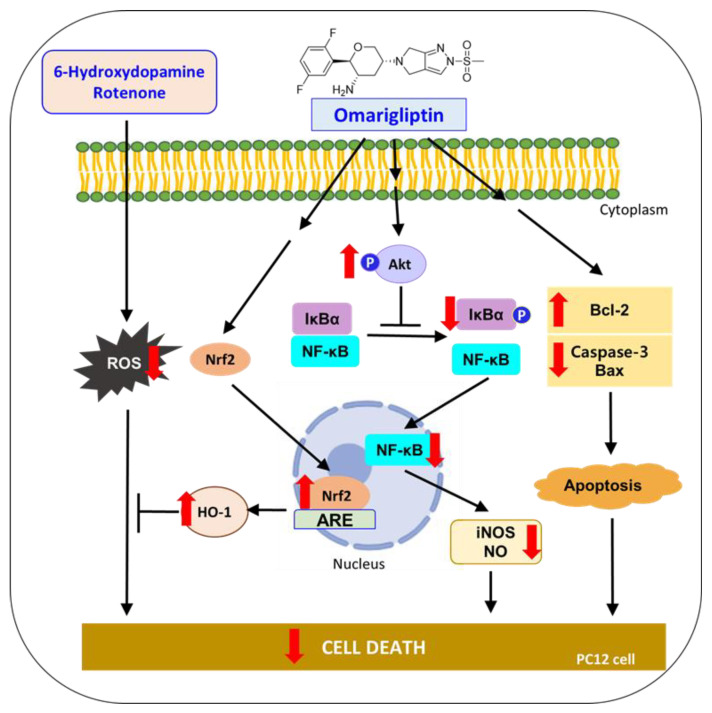
Suggested molecular mechanisms underlying the neuroprotective effects of OMG in PC12 cells. 6-OHDA, 6-hydroxydopamine; ROS, reactive oxygen species; Nrf2, nuclear factor erythroid 2–related factor 2; HO-1, heme oxygenase-1; IκBα, inhibitory kappa Bα; NF-κB, nuclear factor-kappa B; iNOS, inducible nitric oxide synthase; NO, nitric oxide; Bcl-2, B-cell lymphoma 2; Bax, Bcl-2-associated X.

Together, these results demonstrate that OMG protects PC12 cells from the oxidative toxicity of 6-OHDA or rotenone. The antioxidant activity of OMG through the activation of the Nrf2/HO-1 pathway, anti-inflammatory activity through the inhibition of NF-κB nuclear translocation, and its anti-apoptotic function of inhibiting pro-apoptotic proteins and enhancing anti-apoptotic molecules are characterized in 6-OHDA- or rotenone-treated PC12 cells. Based on these findings, suggested molecular mechanisms underlying the neuroprotective effects of OMG in PC12 cells are illustrated in Figure 10. All these findings support the therapeutic potential of OMG in the treatment of PD and provide multiple mechanisms of action by which OMG exerts an antiparkinsonian effect. Interestingly, we found that OMG exhibited the same mode of protective activity against the oxidative toxicity induced by two different kinds of neurotoxin with distinct mechanisms. Further study is warranted to clarify potential cross-talks between these signaling pathways and confirm the therapeutic efficacy of OMG in animal models of PD.

## Data Availability

All of the data are contained within the article.
